# Depolymerization of Rice Straw Lignin into Value-Added Chemicals in Sub-Supercritical Ethanol

**DOI:** 10.1155/2022/7872307

**Published:** 2022-05-20

**Authors:** Viet T Tran, Tan M. Le, Phu V. Vu, Hien M. Nguyen, Yen H. P Duong, Phung K. Le

**Affiliations:** ^1^Faculty of Chemical Engineering, Ho Chi Minh City University of Technology (HCMUT), 268 Ly Thuong Kiet Street, Ho Chi Minh City, Vietnam; ^2^Vietnam National University Ho Chi Minh City (VNU-HCM), Linh Trung Ward, Thu Duc District, Ho Chi Minh City, Vietnam

## Abstract

Depolymerization of lignin is an important step to obtain a lignin monomer for the synthesis of functional chemicals. In the context of more lignin produced from biomass and pulp industry, converting real lignin with low purity is still required more studies. In this study, the influence of solvent composition and reaction parameters such as binary solvents ratio, time, and temperature, the solvent-to-lignin ratio on the depolymerization of rice straw lignin was investigated carefully. Essential lignin-degraded products including liquid product (LP), char (solid), and gas were obtained, and their yields were directly influenced by reaction conditions. Results show that the maximum lignin conversion rate of 92% and LP yield of 66% was under the condition of 275°C, 30 min, 75 : 1 (mL solvent/1 g lignin), and ethanol 50%. Gas chromatography-mass spectroscopy (GC-MS) analysis was used for the analysis of the depolymerization products and identified 11 compounds which are mainly phenolic compounds such as 2-ethylphenol, 3-ethylphenol, phenol, methyl 2,4,6-trimethylbenzoate. The structure changes of LP and char in various conditions were analyzed using Fourier-transform infrared (FTIR).

## 1. Introduction

Climate change is one of the most attractive issues of the 21^st^ century. To surmount this issue and other environmental problems requires the transition from fossil fuel to renewable and sustainable resources partly or completely [1]. Lignocellulosic biomass is a valuable and renewable carbon source that can be utilized for producing chemicals, energy, and materials [2]. Among lignocellulosic biomass, rice straw, an agricultural residue, is considered to be a potential and renewable feedstock owing to its global abundance [3]. According to the estimation of the Food and Agriculture Organization (FAO), there are 760 million tons of rice straw produced annually in the world, to which Asians contribute 667 million [4]. About 50% of produced rice straw is commonly burned in the field because it is a convenient way of disposal and possible to provide nutrients for the next crop, however, that caused a significant detrimental influence on the environment as well as human health [5]. Therefore, based on the rice straw structure comprised of three primary carbon-rich components as cellulose, hemicellulose, and lignin, as well as minor traces of inorganic components (silica), there have been many studies on converting cellulose and hemicellulose from rice straw into chemicals and bio-based fuels, for instance, bioethanol, biobutanol [2, 6, 7]. Meanwhile, lignin was considered the by-product of the conversion process of hemicellulose and cellulose without being effectively utilized [8]. In the United States, about 227 billion liters of bioethanol from biomass are needed to produce for replacing 30% of fossil fuels by 2030 [9]. Therefore, there will have about 225 million tons of lignin by-product, equivalent to about 0.5–1.5 kg of produced lignin per liter of ethanol [10]. With the huge reserves, biorefineries are attempting to recover and utilize lignin for increasing profitability or shortening the payback period. Very recently, our team was also developed a simple method to recover lignin with low silica contamination [11]. This technology was scaled up on a pilot scale and integrated with a bioethanol production system by Le et al. [12]. The results showed that the lignin and silica recovery yields from the black liquor of straw-based bioethanol process were respectively 91.5% and 95%. Although the obtained lignin purity in this process is 78.9%, the silica removal is up to 90% and this process has many merits, e.g., simple, and high production yield.

Lignin, the second most abundant biomass after cellulose, is constituted by three phenylpropane structural units including p-coumaryl alcohol, sinapyl alcohol, and coniferyl alcohol, linked with each other haphazardly by carbon-carbon(C-C) or ether bonds (C-O-C) [13]. Owing to the aromatic structure, lignin has a strong potential for producing liquid biofuels and aromatic chemicals such as vanillin, phenol, guaiacol [14, 15]. Likewise, lignin and its derived compounds are used as platform chemicals in polymer material, medicine, fertilizer, and wood preservatives [16]. The valorization of lignin plays an important key role in the development of biorefinery processes for the production of biofuels, biomaterials, and biochemicals [17]. Very recently, Le et al. [18] proved the “green” concept of biorefinery from biomass through depolymerization of lignin by-products. They claimed that converting lignin could create more values and lower solid waste for the bioethanol production process. Furthermore, in a context where the concept of sustainability is increasingly attracting great attention, the development of flexible and integrated biorefineries to produce biofuels and bioproducts from renewable biomass sources has emerged as the top priority for the gradual transition from a petroleum-based economy to a biorefinery-based circular bioeconomy [19, 20]. Therefore, studying the lignin depolymerization procedure, feasibly applied in many paddy-based biorefineries or creating a small loop in a circular bioeconomy concept, is critical and meaningful due to its values and the intrinsic recalcitrance and structural complexity of lignin, especially low purity lignin.

The thermochemical process is a popular method for converting lignin into biofuels with more efficiency in terms of decomposition ability and reaction time [21–23]. However, most thermochemical processes could be separated into three sub-categories: gasification (>900 K), pyrolysis (650–800 K, 0.1–0.5 MPa), and liquefaction (500–600 K, 5–20 MPa) [24]. Therein, liquefaction is an effective method for lignin valorization. Various value-added phenolic compounds are produced in high yields through solvent depolymerization of the lignin due to its high content of aromatic portions in the macromolecule [25]. Morais et al. [26] reported a green biorefinery concept of the wheat straw lignin depolymerization method by imidazole for the production of value-added phenolic compounds such as vanillin. Alongside with lignin source and structure, the solvent is a crucial factor in depolymerization due to its effects on the conversion of lignin and the yield of phenolic monomers and dimers [27]. Many solvents were utilized in lignin depolymerization, the most common of which were water, organic solvents (alcohols, phenols, and aprotic solvents), and co-solvents. Water is a green and inexpensive solvent as a common solvent in the hydrolysis of lignin. This degrading method of C-O-C bonds in lignin results in phenolic compounds and their derivatives with yields of less than 10% [28]. Also, because of their high solubility and lignin breakdown, alcohols like methanol and ethanol are commonly employed as efficient solvents for converting lignin [29]. To possess the merits of each solvent, some co-solvents with distinct properties and functions, such as alcohol-water, aprotic solvents-water, and aprotic solvents-alcohol, were investigated in the effective depolymerization of lignin into phenolics.

The lignin depolymerization was performed under sub- and supercritical conditions of co-solvent exhibited high selectivity and convenience on products and solvent separation. Morais et al. [17] have shown that in addition to good delignification and extraction of lignin, supercritical CO_2_ fluids also demonstrate effectiveness in the depolymerization of lignin. The lignin depolymerization reactions were performed in a CO_2_/acetone/water mixture and the maximal yield of 10–12% was obtained for monomeric compounds such as vanillin, syringaldehyde, and syringol [30]. Apart from the outstanding advantages, current efforts to develop new methods of CO_2_ biomass value have not yet ensured its technical and economic feasibility due to high technology investment costs. Compared to other supercritical solvents like CO_2_, diverse organic chemicals that fulfill requirements of the principles of green chemistry have been evaluated in sub- and supercritical states for use as solvents for depolymerization of lignin due to their non-toxic, simple, widespread, and low cost. Yokoyama et al. [31] studied lignin depolymerization in sub- and supercritical water and found that when water density rises, the yield of oil products containing hydroxyl groups (maximum 40%) rises and the char production (minimum 30%) falls. Further, Cocero et al. [32] also stated that water under supercritical condition exhibit a low dielectric steady, like non-polar chemicals, therefore it may efficiently solubilize a variety of organic molecules such as lignin. Saisu et al. [33] show that the conversion of organosolv lignin into 2-cresol by supercritical water combined with phenol at 400°C for 1h only gives the production yield of 7.15 wt%. In particular, ethanol and methanol were proved the ability for dissolving and settling the lignin-derived high molecular weight in the depolymerization cycle [34]. Cheng et al. [35]. Revealed that utilizing supercritical ethanol for pine sawdust lignin depolymerization at 300°C for 2h was more effective than methanol and converted 89 wt% lignin. Supercritical ethanol also was demonstrated the capacity of weakening the linkage bonds of lignin because of the hydrogen bond donating of solvent, thus degradation of lignin with supercritical ethanol can produce phenolic compounds [36]. Cheng et al. [37] performed the depolymerization of alkali lignin with 50/50 (v/v) water-ethanol at 200°C under 5 MPa H_2_ with or without a catalyst effectively. They found that high temperatures cause char formation and recondensation, meanwhile, low temperature provides insufficient energy to break lignin ether bonds.

With the reserve of rice straw-based lignin, considering the depolymerization of rice straw lignin for functional chemicals is needed to be required. Although the supercritical technique for depolymerization of lignin model compound was studied, the depolymerization of real lignin with low purity has not been reported elsewhere due to its fewer applications. For the first time, the rice straw lignin with low purity was utilized as a feedstock of solvolysis depolymerization. The goal of this study was to investigate the effects of various parameters, such as temperature, reaction time, solvent ratio, and solvent concentration on the distribution of products after depolymerization of rice straw lignin. Ethanol was used as a solvent because of its hydrogen-donor capability, with a relatively low critical point (243°C, 6.39 MPa). The chemical structure of bio-oil and char after the reaction was determined chemical structure by FTIR, and the quantitative analysis of bio-oil was measured by GC-FID combined with an MS detector.

## 2. Materials and Methods

### 2.1. Materials

The rice straw lignin was obtained using the method developed by Do et al. [11]. Briefly, rice straw was soaked with NaOH 1 wt% at 90°C for 2 h. Afterward, the rice straw residue was removed from the mixture to obtain the liquid by a vacuum filter. The obtained liquid was stepwise acidified with H_2_SO_4_ 18 wt% to pH 9 to remove silica, and then, it was continuously adjusted to pH 3 to recover lignin.

The following reagents, solvents, and gasses were purchased from commercial suppliers: sodium hydroxide (NaOH), sulfuric acid (H_2_SO_4_), ethanol (EtOH), ethyl acetate (EA), tetrahydrofuran (THF), hydrogen, and nitrogen.

### 2.2. Depolymerization of Paddy Straw Lignin

All reactions were carried out in stainless steel autoclave batch reactor (4540 Parr reactor – 4848 reactor controller) with a thermocouple, pressure transducer, gauge, jacketed electrical heating, and mechanical agitation device. A total of 2.0 g of lignin and 50 mL of solvent were loaded into the reactor. During the reaction, the mixture was kept stirring at 200 rpm. The accuracy of the temperature controller was within  ± 2°C. The initial temperature of the reactor was 30°C and the heating rate was 5°C/min in all cases. After the reaction, the reactor was cooled to room temperature. The solid residue was separated from the liquid by filtration and then washed with the same solvent used in each reaction to remove any liquid from the solid. EA was used to extract the liquid phase to obtain the EA phase. After the filtration EA phase, the bio-oil/ liquid product (LP) was obtained by vacuum rotary evaporation at 50°C. The solid phase was washed with THF and stirred for 3 hours in a beaker. After filtration of the THF phase, the obtained undissolved solid (char) was oven-dried at 105°C for 24 h. The difference in the weight of solid products after dissolving with THF is the weight of residual lignin. The conversion rate and yield of various products were calculated using the following equation:

(1) $$\begin{array}{c} \text{LP yield}\left(\mathrm{wt}\right)=\frac{\text{weight of oil fraction }\left(g\right)}{\text{weight of initial lignin }\left(g\right)}\;\times \;100\text{\%}. \end{array}$$

(2) $$\begin{array}{c} \text{char yield}\left(\mathrm{wt}\text{\%}\right)=\frac{\text{weight of char }\left(g\right)}{\text{weight of initial lignin }\left(g\right)}\;\times \;100\text{\%}. \end{array}$$

(3) $$\begin{array}{c} \text{gas yield}\left(\mathrm{wt}\text{\%}\right)=\text{100}-\left(\text{oil yield}+\text{char yield}\right)\text{.} \end{array}$$

(4) $$\begin{array}{c} \text{conversion rate}\left(\text{\%}\right)=\frac{\text{weight of initial lignin }\left(\text{g}\right)\text{–weight of residual lignin }\left(\text{g}\right)}{\text{weight of initial lignin }\left(\text{g}\right)}\;\times \;100\text{\%}. \end{array}$$

### 2.3. Analysis of Lignin and Biocrude

The infrared (IR) spectra of lignin, biocrude, and char were acquired using an FTIR spectrometer (PerkinElmer Frontier FTIR) to determine the chemical structure of the samples. The biocrude is analyzed by GC-FID-MS on Agilent 6890N GC with a DP-5 column and DP-5 MS column. A volume of 0.5 *μ*L was injected for each sample, and the inlet temperature is 280°C. The temperature procedure was as follows: 60°C (4 min) to 150 °C (rate of 10°C/min, hold in 5 min), finally to 280°C (rate of 20°C/min, hold in 10 min). Nitrogen served as the carrier gas. The identification of organic molecules in the bio-oils was accomplished by matching the data in the NIST library.

### 2.4. Statistical Analysis

All analyses were done at least in triplicate, and the data are expressed as mean values ± standard deviation (S.D.) for each measurement.

## 3. Results and Discussion

### 3.1. Lignin Characterization

**Figure 1:**
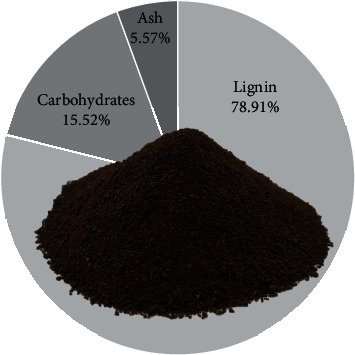
The physic and composition of rice straw lignin.

In this study, lignin was recovered from rice straw following the method of our team with a recovery yield of 52.64% [11]. The obtained lignin (Figure 1) is a dark brown powder with a purity of 78.91% determined by the method of NREL under the number NREL/TP-510-42618 [38]. The composition of lignin obtained from straw is including 78.91% pure lignin, 15.52% carbohydrate content, and 5.57% ash content. Carbohydrate is the remained hemicellulose and cellulose and the ash is mainly silica [12].

**Figure 2:**
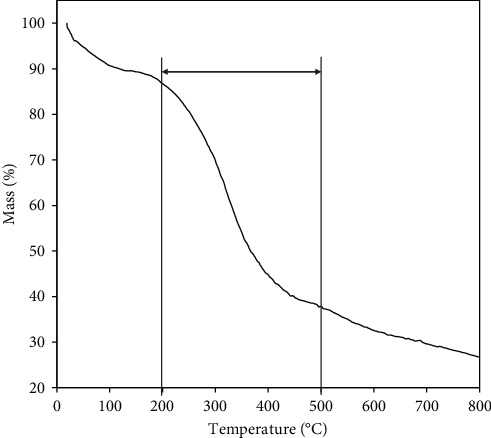
The TG analysis of rice straw lignin.

Figure 2 depicts the mass loss of rice straw lignin over a wide temperature range of 23°C to 700°C, consisting of three stages: dehydration stage (30°C–200°C), rapid degradation stage (200°C–500°C), and slow degradation stage (500°C–700°C). In the dehydration stage, the mass loss of 14.11% was mainly a consequence of the evaporation of moisture and a part of carbohydrates. The mass loss of the rapid degradation stage was caused by the decomposition of the main lignin structure that lead to 59.59% of the lignin mass being transformed into volatiles [39]. This stage was divided into two small stages: the primary stage corresponded to the release of a large amount of volatiles, particularly phenols, while the second stage was related to the increased release of low-molecular-weight volatiles such as CO, CH_4_, CO_2_ because of the secondary cracking of higher molecular weight volatiles [40]. These consequences show that the main degradation reaction of the straw lignin is performed at a temperature range between 200 and 500°C. Many studies also confirmed the lignin liquefaction process in organic solvents as anthracene oil, alcohols, and acetone normally operated at a mild temperature (250–450°C) and long residence time (10–60 min) in organic solvents such as anthracene oil, alcohols, and acetone [41]. After 500°C, the slowly degraded stage was performed in which lignin residue decomposed and transformed into ash and coke eventually.

### 3.2. Role of Reaction Parameters on Bio-Oil Yield

The depolymerization of lignin is a complicated process, in which many reactions happened in parallel. The reaction time, temperature, ethanol concentration, and lignin to liquid ratio are key factors of depolymerization. A long reaction time was needed to ensure complete degradation of all ether bonds in lignin, and gradual degradation of the stable C–C bonds, afterward. The long reaction induction period of the intermediates enabled secondary reactions including repolymerization, crosslinking, and rearrangement [42]. It can result in increased gas or solid product (char) and affect the quantity and quality of oil products. According to S.Kang et al., the temperature of lignin depolymerization reaction condition is over 250°C [43]. The role of heat affects the pressure in the reactor, it can lead to the result that the medium state is changed at each temperature. Besides, the temperature must be suitable for producing the target product, the temperature is low which can generate a high quantity of char while the temperature is high which can have a gas production tendency. Hence, the temperature is the essential aspect to investigate the conversion rate as well as the quality of depolymerization. As mentioned before, ethanol and water are green and effective solvents. To evaluate the solvent composition to the yield of depolymerization process and liquid product, the share of ethanol in ethanol-water co-solvent is risen gradually from 0% to 100% (0%, 25%, 50% 75%, 100%). The solvent states corresponding to the ethanol percentage in the reaction medium at 275°C are shown in the following table. When changing the ratio of ethanol in the solvent, it also affects the state of the reaction solvent, subcritical or supercritical (see Table 1). Therefore, the created products have different quantities and qualify. The solvent state is defined via the comparison of the critical temperature and critical pressure to real corresponding values [44]. As the research of J.Y Kim et al., the solvent and lignin ratio is described as the factor affecting the conversion rate as well as the proportion of reaction products [45]. The share of solvent that affects the reaction differently depends on the temperature and others.

#### 3.2.1. Effect of Reaction Time

**Figure 3:**
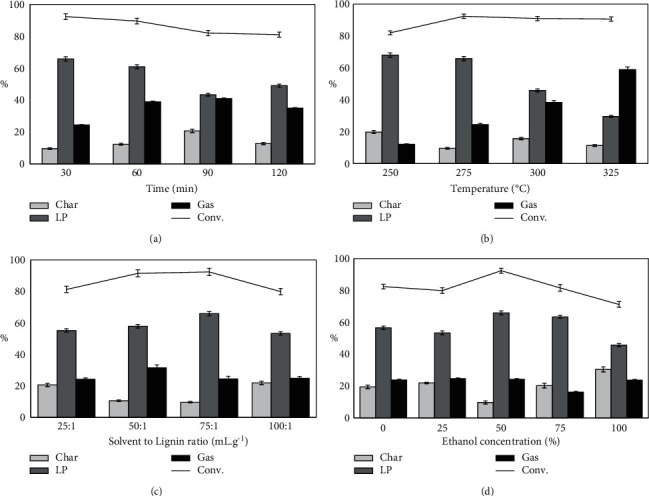
The effects of reaction conditions on the product distribution and conversion rate of rice straw lignin.

Reaction time is an important factor in the product distribution of depolymerization lignin [46]. During the depolymerization of lignin, the yield of biocrude is higher for the short reaction time while the decomposition and repolymerization may be occurred and formed char or gas for too long reaction time. Batch experiments in ethanol 65% were carried out at 250°C and lignin to solvent ratio of 1 : 75 (g:mL) for a reaction time ranging from 15 to 360 min. The influence of reaction time on products of DP lignin is presented in Figure 3(a). As can be seen, the reaction time ranged from 30 to 120 min, the LP yields within a large range of 65.88–43.39% gradually while the quantity of the char increased continuously when the reaction time increased. The yield of LP peaks at 65.88% at 30 min with a conversion rate of 92.39% and the SR yield is almost consistent from 30 min to 120 min until increasing at 180 min. The results are consistent with many previous studies [45, 47]. According to Singh et al. [48], the cracking reaction and condensation occur concurrently during lignin degradation. When most of the linkages in lignin as ether bonds and carbonyl groups were cleaved, the lignin depolymerization was complete after a specific reaction period. The condensation of the degraded lignin intermediates would be favored by extending the reaction time, which is likely why the solid bio-residue yield rose after 30 minutes. As a result, 30 min would be a desirable reaction time for depolymerization of lignin using ethanol.

#### 3.2.2. Effect of Temperature

The reaction temperature is an important factor for product distribution. Raising the temperature would promote the decomposition of lignin and the repolymerization of the intermediates simultaneously. Due to the maximum liquid product yield at 30 min, this reaction time has been considered to investigate the effect of temperature on liquid product yields. The results of lignin depolymerization in different temperature values are shown in Figure 3(b). With the increase in temperature from 250 to 325°C, the yield of LP decreased from 68.08% to 29.64%, whereas, the GP rose significantly from 24.46% to 58.96% because the higher temperature was more promotion for the bond cleavage, especially C-O linkages. Although the liquid yield was maximum at 68.08% at 250°C, the conversion rate has a peak of 92.39% at 275°C. Ye et al. [49] also recorded the same results that the yield of liquid products decreased with increasing temperature from 225 to 300°C for lignin depolymerization in ethanol-water. The depolymerization of lignin was increased following temperature increase, but the higher temperature promoted the secondary reactions, representing further repolymerization of LP to char as well as the LP decomposition to gas as regularly observed [45]. When the reaction temperature rises, the reactive sites in the ortho positions of the phenolic hydroxyl groups are susceptible to condensation with alcoholic side chains, resulting in carbon-carbon bonding and crosslinking to the formation of char [50]. It can be inferred from the above results that the temperature of the reaction can have a drastic impact on the product yield. According to the mechanism proposed by Kang et al. [43], hydrolysis and cleavage of the ether bond and the C–C bond, demethoxylation, alkylation, and condensation reactions occurred, and those main reactions happened to compete in the lignin depolymerization process. Temperature is the most effective parameter influencing the course of the above reactions. When the temperature is sufficiently larger than the activation energies for the bond breakage, extensive biomass depolymerization occurs [50]. In the degradation of lignin and its model compounds, cleavage of the *β*-O-4 ether bond was reported to take precedence, C*α*–C*β* was also an easily broken bond. On the other hand, hydrothermal reactions have little effect on aromatic rings, and biphenyl-type compounds are extremely stable. Thus, phenol monomers and dimers can be produced at low temperatures. Meanwhile, the demethoxylation and alkylation of lignin-derived phenolic compounds would be enhanced as the temperature increased, allowing for the production of a variety of alkylphenols [43]. Along with the increase in temperature, there has been an increase both in the concentration of the free radicals and the probability of repolymerization of fragmented species. Consequently, it is being considered that the temperature has a consecutive effect on the liquefaction products, at first, the increase in the temperature has affected the bio-oil efficiency positively but after the bio-oil efficiency has reached the maximum level, the increase in the temperature has inhibited the bio-oil efficiency [50].

#### 3.2.3. Effect of Solvent to Lignin Ratio

According to Kim et al. [45], the solvent to lignin ratio is described as the factor affecting the conversion rate as well as the proportion of reaction products. In this research, the range of ratio solvent to lignin is investigated at 25, 50, and 75 (mL/g) to assess the oil yield of depolymerization reaction. The yield of LP is proportional to the ratio of solvent volume to lignin mass and it is shown in Figure 3(c). It depicts that as the solvent/lignin ratio increased from 25 : 1, 50 : 1, to 75 : 1 (mL:g), the total yield of LP increased from 55.16%, 57.88%, and 65.88%, whereas the solid (biochar) yield decreased with the increased the solvent/lignin ratio. Besides, the conversion rate of reaction reached a maximum of 92.39% at the solvent/lignin ratio of 75 : 1 (mL:g). All solvolytic conversion methods benefit from the dilution of educts, intermediates, and products during the reaction, in addition to their specific reaction conditions. This dilution reduces cross-reactions between the species involved, resulting in a more focused product range. Increased substrate concentrations invariably result in cross-reactions, which frequently result in polymerization of the reaction products. Hydrothermal biomass liquefaction has also been seen to go through similar processes. High biomass concentrations have been found to encourage the production of solid carbonization products in this scenario [48].

#### 3.2.4. Effect of Ethanol Concentration

The effects of ethanol concentration on the yields of SR and LP from lignin depolymerization at 275°C and 60 min was shown in Figure 3(d). When the ethanol concentration is 50%, the yield of LP and the conversion is the highest, and SR yield is the lowest, which indicates that 50% ethanol could effectively inhibit condensation, gasification, and dehydration of lignin. In pure ethanol and pure water medium, the LP yield is lower, but the LP percentage in the pure water-produced mixture is higher than in pure ethanol. The results are consistent with previous studies by Yuan et al. [51], who suggested that the hydrogen-donor capability of ethanol led to the stabilization of the free radicals generated from lignin depolymerization. Besides, there is less or almost no hydrolysis reaction in pure ethanol solvent, which is the reason explaining why the LP yield produced by pure water is higher. Moreover, the LP mass increased until the ethanol percentage is greater than 50%. It is generally found that ethanol addition enhances the lignin depolymerization because the ether bonds cleavage is performed more easily but the ethanol content increase prevented the solubility of lignin in the reaction medium.

### 3.3. FTIR Analysis for Char and LP under Various Reaction Conditions

**Figure 4:**
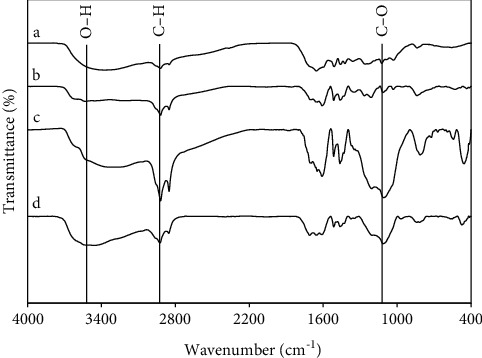
FTIR spectra of rice straw lignin (a) and char/or solid residue from the lignin depolymerization under the ethanol concentration of (b) water, (c) 50%, and (d) 100% at 275°C, 30 min, 75 : 1 (mL ethanol: 1 g lignin).

The FTIR spectra of the raw lignin and LP obtained under different ethanol concentrations were shown in Figure 4 and the characteristic peak of lignin and its depolymerization products in the spectrum was supplied in Table 2. The FTIR profiles of LP were different from that for the rice straw lignin, with some of the adsorption peaks weakened or absented significantly in the former, indicating that some bonds of the alkali lignin were destroyed in the reactions. As can be seen, the transmittance of a peak at 3430 cm^−1^ which presents the hydroxyl group decreased in water and ethanol 50% and increased in ethanol 100% after the depolymerization. Besides, the C-H vibration group, assigned at about 2985 cm^−1^, was stronger following the increase in the ethanol concentration. The reason is that the ethanol can easily generate hydrogen, thus hydrogenation or hydrogen reduction was performed and caused changing of the C-H group [52]. Moreover, the stretching vibration of C-O (1046 cm^−1^) belongs to esters, aldehydes, and ketones, which demonstrate that the LP in this study has the formed compounds due to cellulose and hemicellulose depolymerization.

**Figure 5:**
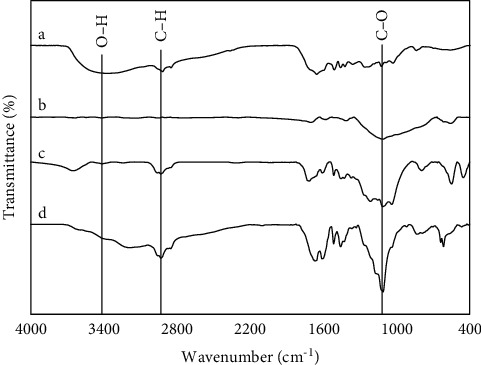
FTIR spectra of rice straw lignin (a) and LP from the lignin depolymerization under the ethanol concentration of (b) water, (c) 50%, and (d) 100% at 275°C, 30 min, 75 : 1 (mL ethanol: 1g lignin).

Figure 5 presents the FTIR spectra of char in different solvents recorded in the range of 4000 cm^−1^ to 400 cm^−1^. According to the infrared characteristic peak data, the peak at 3430 cm^−1^ is corresponded to hydroxy of lignin decreased as low ethanol concentration suggesting a lot of broken-bond of hydroxyl functional groups attached to the aromatic ring was promoted with the decreasing ethanol concentration. The absorption at 2930 cm^−1^ represents the –CH stretch attached to the aliphatic chain and methoxyl group. These bonds increased slightly and vibration peaks were separate in high ethanol concentrations (50% and 100%). The absorptions at 1130 cm^−1^ ascribed to the C-O group were significantly increased in intensity with the rising of ethanol concentration, which might indicate hydroxyl condensation and dehydration occurred at higher concentrations of ethanol. Thus, Kang et al. [43] reported that the char product is formed by homogeneous polymerization of the phenolics and water-soluble compound; on the other hand, non-dissolved lignin undergone heterogeneous pyrolysis and generated char.

### 3.4. GC-MS Analysis

The GC-MS analysis of the liquid product was performed to identify the major low molecular phenols derived from the depolymerization of rice straw lignin. The detailed data for major products of reaction at 250°C, 50% ethanol, solvent to lignin ratio of 75 : 1 (mL:g), and reaction time of 30 min are presented in Table 3. As can be seen, many aromatic compounds are obtained from the depolymerization process, especially 2-ethylphenol, 3-ethylphenol, phenol, methyl 2,4,6-trimethylbenzoate, 1-[4-hydroxy-3-(2-hydroxyethyl) phenyl] ethenone, carvacrol. Zou et al. [53] also reported some products from depolymerization of alkali lignin in supercritical such as 2-ethylphenol, 1-[4-hydroxy-3-(2-hydroxyethyl)phenyl]ethanone. That can be explained that the *β*-O-4 linkage is more easily degraded under moderately mild conditions because of its low bond dissociation enthalpies compared to those of several carbon-carbon bonds [54]. Besides, some open-chain compounds are also determined as 3,6-dimethyl-6-octen-3-ol, 2-O-(2,2-dimethylpropyl)-1-O-hexyl oxalate, 3,7-dimethyldecane. Although such compounds could not be expected during lignin depolymerization, Li et al. [52] also verified that the cellulose contaminated in lignin can be hydrolysis to form ester and the retro-aldol condensation also occurred during this process. In addition, the compounds having C–O or C=O bonds may be connected by the dissociation of O–H bonds and the recombination of C–H bonds to form hydrocarbons. Thus, it can demonstrate that rice straw lignin can produce aromatic compounds through depolymerization.

## 4. Conclusions

This study provided direction for the design of depolymerization parameters for high yield LP from rice straw lignin by figuring out the relationship between reaction conditions and product distribution. Rice straw lignin depolymerization studies were carried out with sub- and supercritical ethanol/water solvent under various reaction conditions, and their resulting yields were significantly affected by the reaction conditions. Maximum LP yield of 65.88 wt.% and conversion rate of 92.39% was obtained at 275°C in 30 min under the sub-supercritical ethanol 50% and the solvent-to-lignin ratio of 75 : 1 (mL/g). In the LP, 11 compounds identified by GC-MS are mainly comprised of phenolic compounds. The open-chain compounds as 3,7-Dimethyldecane, 3,6-Dimethyl-6-octen-3-ol were obtained due to the hydrolysis of cellulose.

## Figures and Tables

**Table 1:** The solvent states in reaction conditions [44].

	Ethanol in the reaction solvent				
	0%	25%	50%	75%	100%
Critical temperature (^o^C)	374	317	277	252	241
Critical pressure (MPa)	220	148	97	70	61
Real temperature (^o^C)	275	275	275	275	275
Real pressure (MPa)	62	80	86	92	80
State of solvent	Subcritical			Supercritical	

**Table 2:** FTIR wavenumber and functional groups are present in the rice straw lignin and its depolymerization products.

Wavenumber (cm^−1^)	Functional group and vibration type
3500–3100	-OH stretching
2800–3750	Asymmetric stretching and bending vibrations of C-H groups
2900–2935	-CH stretching
1604–1735	C=O stretching of carbonyl, carboxyl, and acetyl group and of xylans
1510–1605	Aromatic skeletal vibration (C=C) of lignin
1455–1465	-CH_3_O stretching vibration
1046–1130	-CO stretching vibration
800–833	-CH bonds in associated to aromatic rings.

**Table 3:** The obtained compounds in straw-derived lignin depolymerization in conditions of 50% ethanol, 275°C, 30 min, 75 : 1 (mL ethanol: 1 g lignin).

No	Compounds	RT^*∗*^	%Area^*∗∗*^
1	Phenol	6.843	3.59
2	(E)-3-Tridecen-1-yne	6.860	2.35
3	1-[4-Hydroxy-3-(2-hydroxyethyl)phenyl]ethanone	6.968	7.14
4	2,3-Dihydroxybenzoic acid	8.931	8.02
5	2-Ethylphenol	8.954	8.29
6	2-O-(2,2-Dimethylpropyl)-1-O-hexyl oxalate	8.977	9.67
7	3,6-Dimethyl-6-octen-3-ol	9.766	5.63
8	3,7-Dimethyldecane	19.110	8.83
9	3-Ethylphenol	19.442	5.47
10	Methyl 2,4,6-trimethylbenzoate	24.300	3.66
11	Carvacrol	31.235	5.36

^*∗*^RT: retention time (min). ^*∗∗*^Total area was obtained based on the integration of 11 major peaks, without including the small peaks with %Area < 1.5.

## Data Availability

The generated data used to support the findings of this study are included within the article.
